# Managing automation developrent in harmony with the rest of an international company—a QC laboratory manager's perspective

**DOI:** 10.1155/S1463924698000121

**Published:** 1998

**Authors:** Paul Newton

**Affiliations:** GlaxoWellcome Inc. 1011 N. Arendell Av. Zebulon NC 27597 USA

## Abstract

Significant opportunities and challenges are presented when transitioning from managing laboratory automation development of pharmaceutical products at a single site to collaborative management with multiple domestic and international sites. Prior to integrating Glaxo and Burroughs Wellcome about two years ago, each company had expertise in laboratory automation, but neither had a strategy for consistent business-justified laboratory automation. The approach for international harmonization of automation development of pharmaceutical test methods that the integrated company has adopted is presented. Some items to consider before undertaking a company-wide automation development harmonization programme are offered for consideration. Experiences encountered and future planned benefits are discussed.


Journal of Automatic Chemistry, Vol. 20, No. 3 (May-June 1998) pp. 91-94

### Managing automation development in harmony with the rest of an international company—a QC laboratory manager's perspective

**Paul Newton**

*GlaxoWellcome Inc., 1011 N. Arendell Av., Zebulon, NC 27597, USA*

*Significant opportunities and challenges are presented when transitioning from managing laboratory automation development of pharmaceutical products at a single site to collaborative management with multiple domestic and international sites. Prior to integrating Glaxo and Burroughs Wellcome about two years ago, each company had expertise in laboratory automation, but neither had a strategy for consistent business-justified laboratory automation. The approach for international harmonization of automation development of pharmaceutical test methods that the integrated company has adopted is presented. Some items to consider before undertaking a company-wide automation development harmonization programme are offered for consideration. Experiences encountered and future planned benefits are discussed.*

### Introduction

The environment within today's pharmaceutical industry is one of tough, world-wide competition in international markets. For a large, international company like Glaxo-Wellcome to be successful, high efficiency must be obtained through the organization. It is important to reduce the time from clinical trials to the launch of a product. It would be most effective to minimize duplication of effort and maximize standardization of work practices within the company. One way that the analytical chemistry testing areas can gain efficiency is to automate labour intensive or technique-dependent activities. As part of this effort, harmonizing the development of automated test methods should be considered. In order to put this into practice, many activities must be co-ordinated between testing and development laboratories. This task is complicated for a company like GlaxoWellcome by the fact that it has many distant laboratories with different cultures and/or regulatory drivers which are developing methods for the same products. This means that there is a risk that the harmonization process will be slow, frustrating, and non-productive. However, if planned and managed well, appropriate alignment with other analytical groups can proceed smoothly and productively.

### Before establishing the programme at GlaxoWellcome

Since there is more than one group developing laboratory automation for the same products at GlaxoWellcome, it was worth considering harmonization of automated methodology. Before work was started on a standardized company-wide laboratory automation development process, the following items considered:

- What is the potential business value of automated test methods in the company?
- What is the scope of the harmonization plan; which laboratories will be involved?
- What is the magnitude of the overlapping automation needs in other testing groups?
- What efficiencies could be gained by one team co-ordinating the development of automated test methods?
- How flexible can each testing laboratory be regarding the final automation solution?
- Can the same test method be used at all of the sites?
- Is any group 'married' to specific technologies or suppliers?
- What is the cost of transferring non-standard automated test methods to the testing laboratories?
- What is the benefit of having automated test methods available earlier in the product life cycle, prior to NDA stability studies and NDA/MAA registration?
- With existing staff, how long will it take to develop/maintain/enhance specific automated methods at each site independently?
- What are the regulatory compliance benefits of having different laboratories using identical automated test methods on common products?

A business justification was required in order to evaluate if the harmonization effort would be worthwhile. Many assumptions about different items had to be made. The accuracy of those assumptions will greatly influence how close the estimated net benefit will be to the actual benefit. It was therefore important to try to get accurate estimations when building the cost/benefit model for the programme. Some items considered were:

- Number of products tested at multiple sites.
- Number of batches of each product tested at each site.
- Number of methods to be automated for those products.
- Sample flow into the lab.
- Time period required to test manually.
- Analyst's time required to test manually.
- Time period required to test using automation.
- Analyst's time required to test using automation.
- Number of batches that can be tested manually per analyst per day.

0142-0453/98 $12·00 © 1998 Taylor & Francis Ltd

- Percentage of the analyst's time dedicated to manual testing.
- Number of batches that can be tested using automation per analyst per day.
- Percentage of the analyst's time dedicated to automated testing.
- Cost of equipment to test manually.
- Cost of automated equipment.
- Cost of analyst's time.
- Other operating costs such as supplies, service, maintenance.
- Depreciation rate.

There needs to be a clear achievable advantage in investing in a harmonization programme. A broad assessment of the potential net value of the program was provided for management's evaluation. One point that was emphasized was that the standardization process is a significant undertaking, requiring substantial resources to establish and maintain. The costs and benefits of this undertaking were estimated based on assumptions which were our best estimates as to what the business would look like in the future. Benefits include:

- Redundant automation development will be minimized within the operation.
- A superior infrastructure (validation protocols, documentation, etc.) will be developed.
- Consistency of various practices will be maximized across sites which will minimize regulatory compliance issues.
- Efficiency of transfer of automated methods between groups will be maximized.
- Automation solutions will be achieved faster and at the right time.
- Superior automation solutions will be realized.
- Automated solutions will become available which could not be justified at individual sites.
- Education on automation technology and about other areas of the company will increase.
- Contacts to discuss automation problems/opportunities with will increase.
- Agreements on common technology platforms can result in a stronger negotiating position with suppliers.
- The opportunity for staff development will increase.

Potential risks/costs include:

- Conflict may arise between groups ('not invented here', personality conflicts, inadequate agreement up front, different support levels from participants, etc.).
- There may be different priority/timing requirements between groups.
- There may be different preferences in technology and/or suppliers between groups.
- The best technology solution may not be supported at all times.
- The compromises required to harmonize may not be worth the gains.
- The equipment and/or software versions/upgrades may be difficult to co-ordinate.
- The time and costs required to develop/sustain the process may be considerable.
- Team members may be caught between opposite pressures coming from local site business needs and the automation team.
- Sufficient resources may not be available to develop standard infrastructure.
- The automation solutions including hardware and software may be expensive.

Once management support is obtained, the implementation plan with estimated costs and benefits including assumptions should be documented. This document should also contain the commitments from the management that are needed for success.

### Establishing the harmonized laboratory automation programme

Before starting to build a harmonized automation programme, the key players need to agree on a basic goal. The goal was to develop a common process to create efficient, rugged automated test methods using one common development/validation protocol on a common equipment/software platform, which meets the business needs of all of the collaborating testing laboratories. Harmonization does not have to be an all-or-nothing situation. Before starting the standardization effort, each group needed to consider to what degree they wanted to harmonize.

At GlaxoWellcome, an international automation harmonization strategy (IAHS) group was established comprising managers of quality control and development groups from several major sites. A balance between the development and user groups was established, as well as a balance between members from Europe, North America, etc. The members of the IAHS group were all technically oriented, but not currently hands-on testers or developers. This group was responsible for reporting to a more general international science board comprising managers, directors and vice presidents. The initial role of the IAHS group was to establish high level goals, develop a high level strategy, identify automation areas to harmonize and to develop a model to measure the net value of this effort.

There were a number of basic tasks to be considered by the IAHS group when developing the standardized laboratory automation development process. The optimum timing and order of these will vary from organization to organization. Some of the tasks that IAHS group addressed were:

- Present the proposal to targeted laboratory groups to determine their interests.
- Agree on basic goals, assumptions and models to calculate costs and benefits.
- Supply senior management at each site with a justification for the harmonization project.
- Set up communication links.
- Establish a mission statement.
- Identify major automation areas to be addressed.
- Select team members and leaders for each focus area.
- Provide guidance and support to focus teams.
- Monitor and communicate progress of focus teams.

After a mission statement, general objectives and guiding principals were established, several areas of opportunity for laboratory automation development harmonization were identified. For each identified area (solid dosage forms, metered dose inhalers, etc.), focus teams were created to address the standardization of automation development. Each focus team was assigned two sponsors who were IAHS group team members, normally one from the development area and one from the testing area. The role of the IAHS group sponsor is to provide high level direction and to ensure that these focus teams are accomplishing the overall goals of the harmonization effort. Focus team responsibilities included:

- Agree on common technology platforms (hardware and software).
- Agree on how upgrades will be managed.
- Agree on common practices covering: laboratory instrument qualification; method validation protocols and reports; method transfer protocols and reports; acceptance criteria for method validation and transfers.
- Establish communication links between the team members and with the strategy group sponsors.
- Assess the automation needs and resources available to develop automated methods.
- Identify and prioritize specific automation projects using business acceptance criteria.
- Establish milestones and timelines for specific projects.
- Ensure that specific automation projects stay on track.
- Share existing automation solutions.
- Communicate details of automation projects in progress.

The members of the GlaxoWellcome focus teams are a mix of first line supervisors and hands-on scientists from both development and testing laboratories. They will either work on automation projects themselves or direct the work of others who are assigned to the projects. The focus teams have defined the detailed common infrastructure components for harmonized technology. Two co-leaders are assigned for each team so that both the development and quality control areas are adequately represented.

### Our experience to date

The IASH group has been formally meeting since October 1996. This group has representatives from development and quality control in North America, Europe and Japan and meets quarterly with a video conference approximately half-way between meetings. The face-to-face meetings are aligned as much as possible with other quarterly analytical meetings to minimize travel time and expenses. The IAHS team has identified several focus areas and three focus teams are fully functional at this time with significant progress achieved in two areas.

### *Metered dose inhaler (MDI) focus team*

One focus team is working on developing automated test methods for metered dose inhalers. There was an informal international team already in existence before the official automation development harmonization effort was launched. The focus group sponsors agreed that the existing team needed no change in membership in order to meet the IAHS group's objectives. The existing team's goals were aligned well with the company's automation needs, so no adjustments to the team's activities were required. A second chairperson was assigned to this focus team so that both the quality control and development areas were represented in the leadership. This team has only met face-to-face once, shortly after officially forming. They normally hold a video conference meeting every four to six weeks. A common shared area on a universal server has been set up so that information is easily accessible to all members. The IAHS group sponsors keep in touch with the focus team's progress which is communicated to the IAHS group periodically. There are two areas that are being addressed by the metered dose inhaler focus team.

The first analytical application that was addressed had two different automation solutions already far into development by two different groups. Since one of the solutions was a relatively inexpensive partially automated solution and the other was a more expensive fully automated solution, it was decided that both projects would be completed. The focus team has tied up all the loose ends for both projects and equipment is currently being fabricated for several testing laboratories. Although all the sites had influenced both final automated solutions, the day to day running of each project was run by only one group. It was felt that this made each project run more efficiently. It is encouraging to see that sites have already ordered equipment that was developed by the 'other' site.

The second area that is being addressed by the metered dose inhaler focus team was not as far along in development as the first. A requirements document was developed by the focus group and has been distributed to potential manufacturers of the hardware for bidding purposes. The team will select a vendor based on their responses to the requirements document, considering items such as capabilities, service, price, time to delivery, etc. Although this project is currently being managed at only one site, considerable input continues to be provided by several sites. Since there is a high level of trust within the team, this approach seems to be working very well and all team members are satisfied with this mode of operation.

### *Solid dosage form focus team*

Another focus team is concentrating on content uniformity and dissolution analysis of solid dosage forms. This team was established after the formation of the IAHS team. From the beginning, this team has been in alignment with the automation standardization process. Common technical platforms, validation protocols and acceptance criteria have been agreed upon. Since different analytical groups were using different versions of software, a standard version of software has been selected for each type of hardware. Once all participating laboratories have installed the common hardware platform and software version, existing automated methods will be transferred to other testing sites. Their focus team is also currently agreeing on priorities to determine which new or current manual methods will be developed on the common platforms. Each testing lab can decide which of their existing methods will be aligned with the common platforms and which will be left alone.

### The future

When standard equipment platforms and software versions have been installed and new methods are being developed and validated using standard protocols, there will be minimal resources required to maintain the harmonized automation development process. The focus team will only need to meet periodically (about every six months) to consider the value in upgrading hardware, software, or standard protocols as improved technology emerges and regulatory or laboratory requirements change.

When a harmonized approach to automated testing and development is adopted, it will be to the company's advantage to develop automated test methods well before the product goes to market. This will allow the clinical supplies, NDA stability batches, process validation samples and early commercial release and stability batches to be tested efficiently. Also, there should be complete correlation in the assay data from the development stage through the end of the product life. The assay specifications for the product would then also be based on data generated with the same systems that will be used to test samples throughout its product life cycle.

The test methods going into the regulatory submissions will be automated test methods. Any manual testing would be performed with hardware that is equivalent to the automated hardware. For example, if the automated equipment employed a homogenizer for content uniformity samples, manual testing would be performed using an equivalent stand-alone homogenizer instead of the traditional shaker or sonicator. The quality control lab will not have to develop and validate a new set of automated test methods in order to test effectively. This will free up quality control resources by allowing samples to be tested efficiently at the time of production introduction and avoiding lengthy post-launch method improvement projects. The method transfers to quality control at that time will be efficient and straightforward, since the hardware and software platforms will be identical to that on which the method was developed.

### Conclusion

A lot of planning is needed to achieve a harmonized laboratory automation development process in a multisite company. However, if good team players from both development and testing laboratories are involved and this process is supported by upper management, harmonization is achievable. Once the process is in place, a company would have efficient automated test methods that meet the testing laboratory's needs through the product life cycle. These methods would be easily transferred to other testing laboratories in the company. There would no longer be several sets of different test methods being developed and validated for one product in different laboratories. There would be no issues of data not corresponding to data generated in other participating laboratories and the possibility of data not corresponding to the product specifications. At this time, we have enough experience with the automation development harmonization process to have full confidence that the benefits will be well worth the effort.

### Acknowledgements

The author gratefully acknowledges contributions to this paper from Alan Potter, John Glennon, Bill Howard, Pat Henagan, and Gary Coppack, all of GlaxoWellcome.

